# Decreased Antibiotic Susceptibility in *Pseudomonas aeruginosa* Surviving UV Irradition

**DOI:** 10.3389/fmicb.2021.604245

**Published:** 2021-02-03

**Authors:** Hai-bei Li, Ai-ming Hou, Tian-jiao Chen, Dong Yang, Zheng-shan Chen, Zhi-qiang Shen, Zhi-gang Qiu, Jing Yin, Zhong-wei Yang, Dan-yang Shi, Hua-ran Wang, Jun-wen Li, Min Jin

**Affiliations:** Department of Environment and Health, Tianjin Institute of Environmental & Operational Medicine, Key Laboratory of Risk Assessment and Control for Environment & Food Safety, Tianjin, China

**Keywords:** UV irradiation, *P. aeruginosa*, antibiotic susceptibility, oxidative stress, antibiotic resistance genes

## Abstract

Given its excellent performance against the pathogens, UV disinfection has been applied broadly in different fields. However, only limited studies have comprehensively investigated the response of bacteria surviving UV irradiation to the environmental antibiotic stress. Here, we investigated the antibiotic susceptibility of *Pseudomonas aeruginosa* suffering from the UV irradiation. Our results revealed that UV exposure may decrease the susceptibility to tetracycline, ciprofloxacin, and polymyxin B in the survival *P. aeruginosa*. Mechanistically, UV exposure causes oxidative stress in *P. aeruginosa* and consequently induces dysregulation of genes contributed to the related antibiotic resistance genes. These results revealed that the insufficient ultraviolet radiation dose may result in the decreased antibiotic susceptibility in the pathogens, thus posing potential threats to the environment and human health.

## Introduction

In recent decades, antibiotic resistance has emerged as a growingly serious threat to global public health. According to estimates, antibiotic resistance will lead to as many as 10 million casualties annually by 2050 if we don’t take any action at present (O’Neill, 2015). In 2015, the World Health Organization (WHO) announced a global action plan that urges international participants to take immediate action to control and monitor the spread of antimicrobial resistance (WHO, 2015). Hence, antibiotic resistance is receiving increasing global attention (Pruden et al., 2006; Watkins and Bonomo, 2016; Zhu et al., 2017).

In order to control the spread of infectious diseases through the faecal–oral route, disinfection processes, such as chlorination and UV irradiation, are generally applied to kill pathogens during the water treatment (Berry et al., 2006; Gassie and Englehardt, 2017). Although disinfection processes substantially remove antibiotic resistant bacteria (ARB), the general observation is that they cannot completely eliminate antibiotic resistance genes (ARGs; Borjesson et al., 2009; Munir et al., 2011; Huang et al., 2013; Yuan et al., 2014). Accumulating evidence has revealed that chlorination increases the prevalence of ARGs both in and out of bacteria (Khan et al., 2016; Liu et al., 2018; Jin et al., 2020). The emergence of culturable chlorine-injured bacteria that are physiologically unhealthy and suffer from reversible damage due to partial or inappropriate chlorination (McFeters et al., 1986) may play important roles in ARB transmission. Such bacteria were found to be prone to absorbing extracellular ARGs (eARGs) by natural transformation (Jin et al., 2020). This also resulted in enhanced temporary physiological antibiotic resistance to ceftazidime and chloramphenicol in injured *P. aeruginosa* (Hou et al., 2019).

UV irradiation, which is considered a promising physical disinfectant in the water treatment without generating toxic by-products, can penetrate bacterial cell walls and is directly absorbed by nucleic acids, resulting in the inactivation of ARB and damage to ARGs via the formation of dimers of adjacent cytosines or thymine (Zhang et al., 2019). Considering its principal difference from the chlorination process, the UV-induced response in the bacteria may be quite different from the chlorine-induced response. A recent study revealed that UV exposure can lead to the enrichment of bacteria resistant to sulfadiazine, vancomycin, rifampicin, tetracycline and chloramphenicol due to the microbial selectivity of UV exposure to ARB (Guo et al., 2013). In addition, UV exposure strongly stimulates the ability of *Legionella pneumophila* to take up and integrate exogenous DNA (Charpentier et al., 2011). In contrast, a 1 mJ/cm^2^ UV dose can significantly decrease the conjugative transfer frequency of the surviving bacteria (Guo and Kong, 2019). However, few studies have comprehensively investigated the response of bacteria surviving UV irradiation to the environmental antibiotic stress.

In the present study, susceptibility to a panel of six antibiotics was explored in *P. aeruginosa* surviving UV irradiation. The mechanism of antibiotic susceptibility was further investigated by global transcriptional analyses, quantitative real-time PCR (qRT-PCR) validation and antioxidant measurement. The results serve to advance our understanding of the adaption of such pathogens, which can survive UV irradiation, to diverse environments.

## Materials and Methods

### Bacterial Strains and Antibiotics Used in the Study

The *P. aeruginosa* strain was obtained from the American Type Culture Collection (ATCC 27853). Liquid cultures of *P. aeruginosa* were grown in LB medium [10 g/L tryptone (Difco, Detroit, MI, United States), 10 g/L NaCl and 5 g/L yeast extract (Difco)] at 37°C for 12 h. Cetrimide agar (BD Diagnostics, Franklin Lakes, NJ, United States) is a selective medium used for quantitative determination of *P. aeruginosa*. All viable bacteria in the samples were detected by TSYA, a repair medium for injured bacteria (Hou et al., 2019). In general, 1 L of TSYA contains 15 g agar (Oxoid, Basingstoke, United Kingdom), 3 g yeast extract (Oxoid) and 30 g trypticase soy broth (TSB, BD Diagnostics). All antibiotics, including tetracycline, ciprofloxacin, polymyxin B, ceftazidime, chloramphenicol and gentamicin, were purchased from Sigma-Aldrich (St. Louis, MO, United States). Fresh antibiotic solutions were prepared from powder stocks on a weekly basis, stored at -20°C and filter sterilized before use.

### UV Exposure Experiment

A 100 μL suspension of overnight cultured *P. aeruginosa* in LB medium was transferred into another 40 mL fresh LB medium and cultured overnight at 37°C, with shaking at 150 rpm. Bacteria were harvested by centrifugation at 8,000 rpm for 10 min and then washed thrice with phosphate-buffered saline (PBS, pH 7.2), and suspended in 40 mL PBS (the final concentration of *P. aeruginosa* was ∼10^9^ CFU/mL). For UV irradiation, each 40 mL sample was placed onto a 90-mm-diameter petri dish. A low-pressure (LP) collimated beam apparatus containing an LP (120 W, 30% UVC, TL 120W/01, Philips, Holland) mercury UV lamp was used to perform the UV exposure process. The wavelength of the UVC lamp is 254 nm. The corresponding disinfection apparatus were described in more detail in a previous publication (Guo et al., 2013). The exposure time was changed for various plates to reach UV doses of 5.50, 8.25, 11.00, and 13.75 mJ/cm^2^, with irradiance values fixed at 0.275 mW/cm^2^. The exposed samples were preserved for microbial analysis. All the experiments were conducted in triplicate.

### Numeration of the Injured and Total Viable *P. aeruginosa*

The number of viable injured bacteria in the UV-exposed samples was determined according to a previously reported method (Wang et al., 2017; Hou et al., 2019). TSYA medium was used for counting the total number of *P. aeruginosa*, while cetrimide agar plates used for the number of uninjured *P. aeruginosa*. After incubating at 37°C for 48 h, different counts between TSYA and cetrimide agar plates were taken as reference values for the detection of injured *P. aeruginosa*. The lethality rate of *P. aeruginosa* and the percentage of injured bacteria were calculated according to Eqs 1 and 2, respectively:

(1) $$\mathrm{Lethality}\mathrm{rate}\left(\%\right)=\frac{\text{A}-B}{\text{A}}\times 100\%$$

(2) $$\mathrm{Injured}\mathrm{bacteria}\left(\%\right)=\frac{\text{B}-C}{\text{B}}\times 100\%$$

Here, A, detected on TSYA, is the initial concentration of viable *P. aeruginosa* before UV exposure; B, detected on TSYA, is the concentration of viable *P. aeruginosa* following various UV doses and C is the concentration of uninjured *P. aeruginosa*, as detected on cetrimide agar.

### Minimum Inhibitory Concentration Assays

After the UV irradiation, we immediately tested the antibiotic susceptibility of the UV-exposed *P. aeruginosa* using a panel of six antibiotics. Antibiotic minimum inhibitory concentration (MIC) assays were performed using the broth microdilution method according to the Clinical and Laboratory Standards Institute guidelines (Cockerill, 2010). The treated bacteria in the UV exposure experiment were diluted 1:1,000 into fresh Mueller-Hinton (MH) broth (Solarbio, Beijing, China). The untreated *P. aeruginosa* were diluted 1, 000-, 10, 000-, 100,000-fold respectively, making it certain that control group was comparable to UV-treated group. To each cell, 135 μL of diluted bacterial suspension and 15 μL of serial twofold dilutions of antibiotics were added. For the positive control, wells were prepared by inoculating bacteria without antibiotics. A non-inoculated well was used as the negative control. The plates were sealed using an air-permeable membrane (Breathe-Easy, Diversified Biotech, Boston, MA, United States) and incubated at 37°C for 24 h. Then, the OD_600_ of each plate was measured using a microplate reader (BioTek, Shanghai, China), and MIC values were determined as previously described (Hou et al., 2019). All the tests were performed at least three times.

### Measurement of the Activity of Antioxidant Enzymes

*P. aeruginosa* cell samples, at the indicated times following UV exposure, were prepared to assay the oxidative stress response. After sonication at 20 kHz (150 W) for 10 min to break down the bacteria using an ultrasonic cell cracker (VCX750, Sonics, Newtown, CT, United States), the collected samples were centrifuged at 5,000 × *g* at 4°C for 3 min. The levels of superoxide dismutase (SOD), catalase (CAT) and glutathione peroxidase (GSH-Px) in the supernatant were assessed using kits obtained from the Nanjing Jiancheng Bioengineering Institute (Nanjing, China), following the manufacturers’ instructions. The absorbance values of the indicators CAT, GSH-Px, and SOD were determined at 405, 412, and 550 nm, respectively, using a plate reader (BioTek). All the assays were performed in triplicate.

### Total RNA Isolation and Whole Transcriptome Sequencing

*P. aeruginosa* cells exposed to a 11.00 mJ/cm^2^ UV dose were isolated using the Total RNA Isolation Kit (EZ-10 Spin Column, BBI Life Sciences, Shanghai, China), according to the manufacturer’s instructions. Purity of total RNA was determined as 260 nm/280 nm absorbance ratio with expected value of 1.8–2.0 by the GeneQuant 1300 spectrophotometer (GE Healthcare, Milan, Italy). Total RNA was treated with DNase I (Ambion, Grand Island, NY, United States) to eliminate residual genomic DNA. Whole transcriptome sequencing was performed as described previously (Hou et al., 2019). The *P. aeruginosa* PAO1 genome (Genbank accession no. NC_002516.2) was used as a reference for annotation. Genes with fold change (FC) ≥ 2 were recruited as differentially expressed genes (You et al., 2018).

### qRT-PCR Assays

cDNA was synthesized from 500 ng of total RNA using the PrimeScript RT Reagent Kit (Takara, Dalian, China), according to the manufacturer’s instructions. The qRT-PCR assays were performed on the ViiA^TM^ 7 Real-Time PCR System (Applied Biosystems, Foster City, CA, United States) using FastStart Universal SYBR^®^ Green Master (Roche Diagnostics GmbH, Mannheim, Germany). The 16S rRNA was employed as a reference for normalization. The relative mRNA expression levels were calculated using the comparative ΔΔCt method (2^–Δ^ ^Δ^ ^Ct^). The primer sequences are described in Supplementary Table 1.

### Statistical Analyses

Statistical analyses were performed using Microsoft Excel (Microsoft, Redmond, WA, United States), GraphPad Prism and SPSS 19.0. Spearman’s rank correlation test was used to analyze the potential correlations between bacterial composition and MICs of antibiotics. Student’s *t*-test was used to compare quantitative data between groups. *P* < 0.05 was considered statistically significant.

## Results

### Inactivation Curves of *P. aeruginosa* During UV Exposure

**FIGURE 1:**
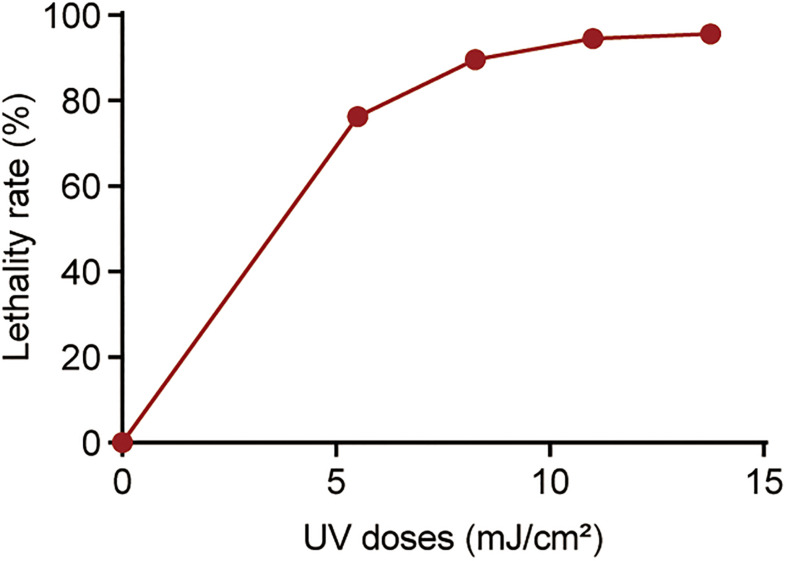
Percentage of killed bacteria following UV exposure. The baseline conditions were as follows: the initial concentration of *P. aeruginosa* was ∼10^9^ CFU/mL (pH 7.2, 20°C). Data are shown as the mean ± standard deviation (s.d.). (n = 3).

To observe the dynamics of bacterial killing, the inactivation curves of *P. aeruginosa* following UV exposure were investigated during bench-scale inactivation experiments (Figure 1). The inactivation curves had two phases: an initial phase with faster kinetics and a subsequent phase with slower kinetics. The initial phase had a clear dose effect, with the inactivation rate increasing with the dose. More than 70% of cells were killed at a UV dose of 5.50 mJ/cm^2^. Subsequently, nearly 94% of cells were killed at a UV dose of 11.00 mJ/cm^2^. No significant increase was noted in bacterial inactivation, even when the UV dose was up to 13.75 mJ/cm^2^.

### Antibiotic Susceptibility of *P. aeruginosa* Surviving UV Irradiation

**FIGURE 2:**
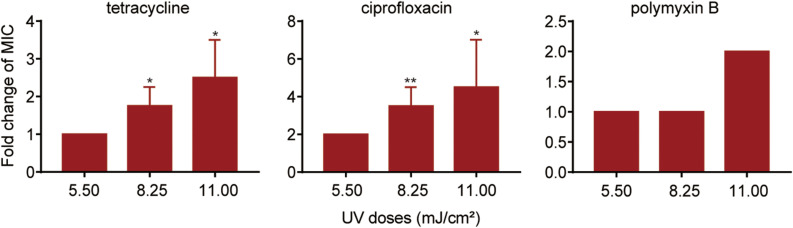
Fold changes in MICs of antibiotics against *P. aeruginosa* surviving UV irradiation, relative to the untreated controls. MIC assays of tetracycline, ciprofloxacin and polymyxin B were performed using the broth microdilution method in accordance with the Clinical and Laboratory Standards Institute guidelines. Data are shown as the mean ± standard deviation (s.d.). (*n* = 4). *P < 0.05 and **P < 0.01; assessed by Student’s *t*-test.

To investigate the effect of UV exposure on antibiotic susceptibility in surviving *P. aeruginosa*, the antibiotic susceptibility of UV-exposed *P. aeruginosa* was tested using a panel of six antibiotics. The results revealed that the MICs of tetracycline, ciprofloxacin and polymyxin B increased quite significantly when the irradiation dose increased from 5.50 to 11.0 mJ/cm^2^; however, no significant increase in ceftazidime, chloramphenicol or gentamicin resistance was observed (Figure 2, Supplementary Table 2 and Supplementary Figure 1). Compared to untreated cells, the MIC of ciprofloxacin against surviving *P. aeruginosa* increased by an average of twofold following exposure to a 5.50 mJ/cm^2^ UV dose. The MIC values of tetracycline and ciprofloxacin increased by 1.8- and 3.5-fold when the UV dose was increased to 8.25 mJ/cm^2^ and by 2.5- and 4.5-fold when it was increased to 11.00 mJ/cm^2^. Moreover, at a UV dose of 11.00 mJ/cm^2^, the MIC of polymyxin B began to increase consistently, up to twofold, against surviving *P. aeruginosa*. Collectively, the results revealed that UV exposure results in a moderate decrease in antibiotic susceptibility in *P. aeruginosa* perhaps through so-called pleiotropic effects.

#### Detection of UV-Injured *P. aeruginosa* and the Effect on UV Injury on Antibiotic Susceptibility

**FIGURE 3:**
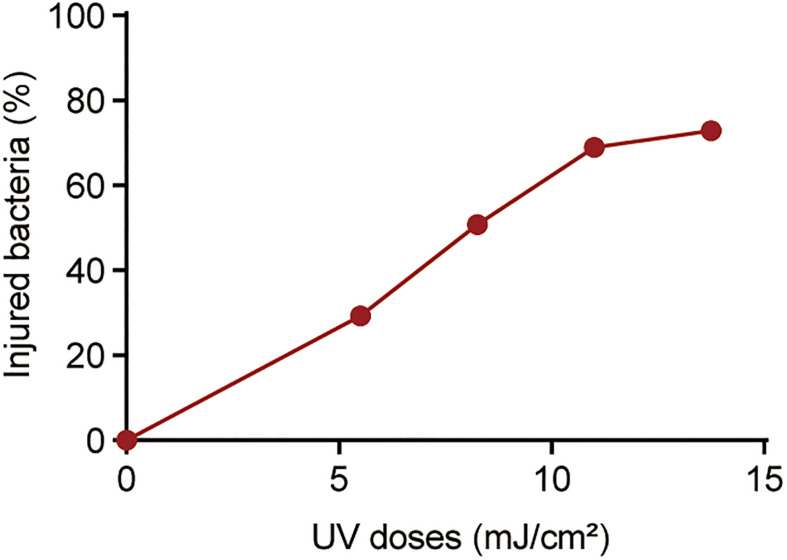
Percentage of injured bacteria following UV exposure. The baseline conditions were as follows: the initial concentration of *P. aeruginosa* was ∼10^9^ CFU/mL (pH 7.2, 20°C). Data are shown as the mean ± standard deviation (s.d.). (n = 3).

Previously, we found that chlorine-injured *P. aeruginosa* showed enhanced temporary physiological antibiotic resistance to ceftazidime and chloramphenicol (Hou et al., 2019). To investigate the contributors to increased MICs of antibiotics in *P. aeruginosa* surviving UV irradiation, the emergence of injured *P. aeruginosa* was investigated (Figure 3). Similar to chlorination, UV exposure led to the constant occurrence of injured *P. aeruginosa*. The percentage of injured *P. aeruginosa* in the viable bacteria increased gradually, and ultimately, almost 70% of the surviving populations with stronger UV tolerance were injured at a UV dose of 11.00 mJ/cm^2^. Their injury curves gradually flattened on increasing the UV dose. However, on analyzing the potential correlations between bacterial composition and MICs of antibiotics, we found no significant correlation between the injury of *P. aeruginosa* and reduced susceptibility to tetracycline, ciprofloxacin and polymyxin B (Table 1). Therefore, unlike *P. aeruginosa* injured during chlorination, the injury of *P. aeruginosa* on UV exposure may not contribute to decreased antibiotic susceptibility.

**TABLE 1:** Spearman’s correlations between bacterial composition during UV exposure and MICs of antibiotics against them.

Lethality rate (%)	Composition	Tetracycline	Ciprofloxacin	Polymyxin B
>50	Injured	0.177	−0.383	−0.866
	Uninjured	–0.265	−0.276	−0.186
≤50	Injured	–	–	–
	Uninjured	–	–	–

#### Antibiotic Susceptibility and Differentially Expressed Genes Induced by UV Irradiation

To reveal the molecular mechanisms underlying the decreased antibiotic susceptibility in *P. aeruginosa* surviving UV irradiation, global transcriptional analyses were performed. In total, 686 genes, including 472 up-regulated genes and 214 down-regulated genes, were found to be dysregulated following exposure to a 11.00 mJ/cm^2^ UV dose (Supplementary Table 3). Eleven up-regulated genes may play a role in decreasing antibiotic susceptibility in *P. aeruginosa* (Supplementary Table 4). This involved the *mexC* gene encoding an accessary membrane protein of resistance-nodulation-division (RND) MexCD-OprJ multidrug efflux pump. The expression level was up-regulated by 2.03-fold (Supplementary Table 4).

To further validate whether *mexC* gene was overexpressed in *P. aeruginosa*, the expression level of *mexC* was monitored by qRT-PCR during exposure to several doses of UV (Table 2). We observed that the expression of *mexC_1_,_2_* was increased by 1.4-fold at a UV dose of 8.25 mJ/cm^2^ but remained at the near-control level at UV doses of 5.50 and 11.00 mJ/cm^2^. Additionally, although *mexC_3_,_4_* was up-regulated at all UV doses used in the present study, the maximum up-regulation, up to 2.83-fold, was observed with a UV dose of 8.25 mJ/cm^2^. MexC is a component of MexCD-OprJ, which is a multidrug efflux pump from the RND family (Poole et al., 1996). The MexCD-OprJ pump functions as a determinant of antimicrobial resistance to several clinical antimicrobials (Poole et al., 1996; Poole, 2005), disinfectants (Morita et al., 2003; Fraud et al., 2008), and other chemicals (Li et al., 1998; Srikumar et al., 1998) in *P. aeruginosa*. These observations indicate that the MexCD-OprJ pump may reduce antibiotic susceptibility in *P. aeruginosa* surviving UV irradiation. It is important to note that the reduced susceptibility is due to the combined action of differentially expressed genes induced by UV irradiation in *P. aeruginosa*. Many factors including MexCD-OprJ, contributed to the decreasing susceptibility in *P. aeruginosa* surviving UV irradiation.

**TABLE 2:** Changes in relative expression levels of select *P. aeruginosa* genes during UV exposure.

Genes		Relative expression levels		

	UVdose(mJ/cm^2^)	5.50	8.25	11.00
*mexC_1_,_2_*		1.2	1.4	1.0
*mexC_3_,_4_*		1.6	2.8	1.7

### Oxidative Stress in *P. aeruginosa* During UV Exposure

**FIGURE 4:**
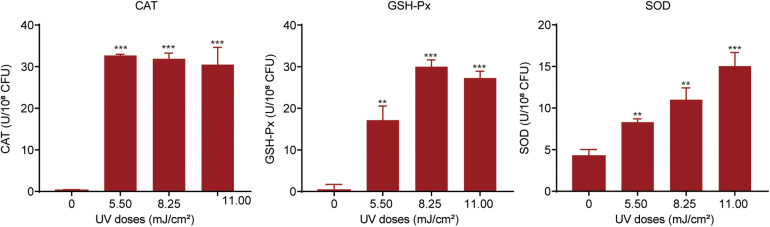
Oxidative stress response in *P. aeruginosa* during UV exposure. Bacterial suspensions were sonicated at 20 kHz for 10 min and then centrifuged at 5,000 × *g* at 4°C for 3 min. The levels of catalase (CAT), glutathione peroxidase (GSH-Px) and superoxide dismutase (SOD) in the supernatants were measured. Data are shown as the mean ± standard deviation (s.d.). (*n* = 3). **P < 0.01 and ***P < 0.001; assessed by Student’s *t*-test.

For protection against oxidative attack, bacteria can alleviate ROS levels by activating or increasing the expression of cellular antioxidant systems (Bae et al., 2011). We investigated the oxidative stress response in UV-exposed *P. aeruginosa*. A strong cellular antioxidant response was observed in UV-exposed *P. aeruginosa*, in addition to a significant increase in the expression of CAT, GSH-Px, and SOD (Figure 4). The CAT level was dramatically increased, by 98.35–105.42-fold, at UV doses of 5.50, 8.25, and 11.00 mJ/cm^2^. However, there was no apparent difference among these three dose groups. GSH-Px activity was up-regulated by 43. 95-, 76. 89-, and 64.95-fold on exposure to UV doses of 5.50, 8.25, and 11.25 mJ/cm^2^, respectively. Obviously, the 8.25 mJ/cm^2^ UV dose resulted in maximal GSH-Px activity. Moreover, the SOD level dramatically increased as the UV dose increased within the observed range. It increased by 1. 95-, 2. 59-, and 3.54-fold on exposure to UV doses of 5.50, 8.25, and 11.25 mJ/cm^2^, respectively. These results were further confirmed by the transcription of antioxidant enzymes. mRNA expression levels of the catalase genes *katB* and *katE* were up-regulated by 3.13- and 2.97-fold, respectively, after exposure to a UV dose of 11.00 mJ/cm^2^. Parallelly, the expression level of *sodM* was up-regulated by 3.29-fold (Supplementary Table 5).

## Discussion

Bacteria that can survive in adverse environments are receiving increasing global attention. In the present study, we found the reduced susceptibility to tetracycline, ciprofloxacin and polymyxin B in *P. aeruginosa* that survived UV irradiation. Mechanistically, we found that UV exposure causes oxidative stress in *P. aeruginosa* and thereby induces dysregulation of genes contributed to the related ARGs, which potentially decreases antibiotic susceptibility in this bacterium. These results reveal that the survival *P. aeruginosa* following UV exposure can survive in adverse environments, including antibiotic exposure, and thus pose lasting hazards to human health. This sheds light on the novel role of UV exposure in the reduction of antibiotic susceptibility in *P. aeruginosa*.

Our findings revealed that the elevated expression of MexCD-OprJ in UV-exposed *P. aeruginosa* may play a role in contributing to reduced susceptibility of antibiotics belonging to the substrates of the MexCD-OprJ pump such as ciprofloxacin and tetracycline (Masuda et al., 1995, 1996). It is of note that the susceptibility of UV-exposed cells to chloramphenicol, a known substrate of MexCD-OprJ, was not affected. Moreover, the reduced susceptibility to polymyxin B at the higher dose of UV irritation cannot be explained by MexCD-OprJ as polymyxins are not considered as the substrates of MexCD-OprJ and act as outer membrane permeabilizers. However, since the increased expression of *mexC* after UV exposure is quite limited, other mechanism(s) may also be possibly involved. A total of 686 genes were found to be dysregulated in UV-exposed *P. aeruginosa*. Among them, the related ARGs including *mexC*, potentially play a combined role in the reduced antibiotic susceptibility. Undeniably, the mechanism(s) affecting antibiotic susceptibility in *P. aeruginosa* remain elucidated, and need to be investigated in the future.

Recent studies indicate that the presence and level of ROS during antibiotic treatment can increase antibiotic lethality (Dwyer et al., 2007; Grant et al., 2012; Kottur and Nair, 2016; Liu et al., 2016), affect the survival of persisters (Grant et al., 2012; Wu et al., 2012) and contribute to the development of drug resistance (Kohanski et al., 2010). Furthermore, increasing evidence has shown that ROS induces the expression of multidrug efflux systems, thereby promoting antibiotic resistance in *P. aeruginosa* (Fraud and Poole, 2011). We have provided evidence that UV exposure increases the expression of cellular antioxidant systems, including CAT, GSH-Px, and SOD, thereby alleviating the ROS levels for protection against an oxidative attack. Collectively, these results indicate that UV-induced oxidative stress may contribute to decreased antibiotic susceptibility in the survival *P. aeruginosa* potentially through the induction of the MexCD-OprJ multidrug efflux pump and other unidentified mechanisms.

In summary, UV irradiation decreases physiological antibiotic susceptibility in the survival *P. aeruginosa*. It may help bacteria adapt to or survive in adverse environments. This also raises the concern that UV disinfection under insufficient dosage to kill all bacteria may pose a potential risk by reducing bacterial antibiotic susceptibility in the environment.

## Data Availability Statement

RNA-seq data have been deposited in the ArrayExpress database at EMBL-EBI (www.ebi.ac.uk/arrayexpress) under accession number E-MTAB-9613.

## Author Contributions

MJ, JL, and HL conceived and designed the experiments and contributed reagents, materials, and analysis tools. AH performed the experiments. TC, DY, ZC, ZS, ZQ, JY, ZY, and HW participated in field research and initial treatment of the materials. MJ, HL, ZC, and SS contributed to the writing and editing of the manuscript. All authors contributed to the article and approved the submitted version.

## Conflict of Interest

The authors declare that the research was conducted in the absence of any commercial or financial relationships that could be construed as a potential conflict of interest.
