# Supplementary material for: Decreased Antibiotic Susceptibility in Pseudomonas aeruginosa Surviving UV Irradition
Source: Front Microbiol. 2021 Feb 3;12:604245. doi: 10.3389/fmicb.2021.604245 (PMC7886673; doi:10.3389/fmicb.2021.604245)
Supplement: Supplementary file 1 [file Data_Sheet_1.docx]

**Supplementary Material**

**Decreased antibiotic susceptibility in *Pseudomonas aeruginosa* surviving UV irradition**

Hai-bei Li^1^, Ai-ming Hou^1^, Tian-jiao Chen, Zheng-shan Chen, Dong Yang, Zhi-qiang Shen, Zhi-gang Qiu, Jing Yin, Zhong-wei Yang, Dan-yang Shi, Hua-ran Wang, Jun-wen Li^*^, Min Jin^*^

Department of Environment and Health, Tianjin Institute of Environmental & Operational Medicine, Key Laboratory of Risk Assessment and Control for Environment & Food Safety, Tianjin, 300050, China

^1^These authors contributed equally to this work.

* **Correspondence**: [jinminzh@126.com](mailto:jinminzh@126.com); junwen9999@hotmail.com

# Supplemental Information:

- Supplemental Figure 1
- Supplemental Tables 1-5

# SUPPLEMENTARY FIGURES

**Fig. S1.** **Fold changes in MICs of antibiotics against UV-exposed *P. aeruginosa*.** MIC assays of ceftazidime, chloramphenicol and gentamicin, were performed using the broth microdilution method in accordance with the Clinical and Laboratory Standards Institute guidelines. Data are shown as the mean ± standard deviation (s.d.).


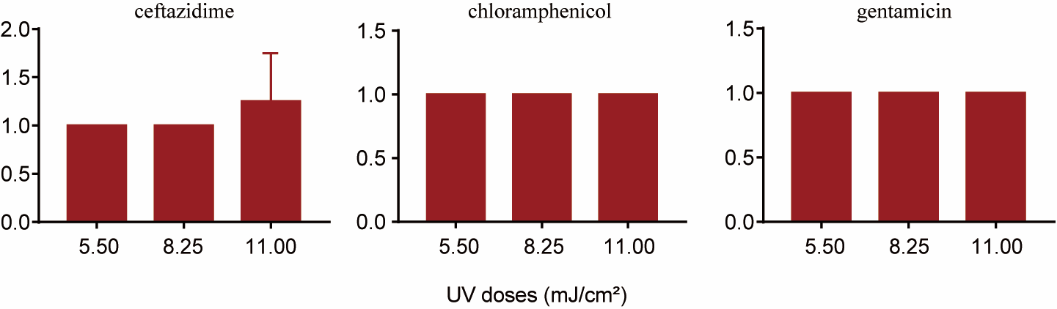


# SUPPLEMENTAL TABLES

**Table S1. qRT-PCR primers for quantification of differentially expressed genes.**

**Table S2. MICs of antibiotics against *P. aeruginosa* surviving UV irradiation.**

**Table S3. Differentially expressed genes in *P. aeruginosa* on UV exposure.**

**Table S4. Summary of changes in gene expression probably related to antibiotic susceptibility in UV-exposed *P. aeruginosa*.**

**Table S5. Changes of gene expression associated with oxidative stress in *P. aeruginosa* following UV exposure.**

**Table S1. qRT-PCR primers for quantification of differentially expressed genes**

| Name | Sequences (5’→3’) | Usage |
| --- | --- | --- |
| *mexC_1,2_*-F | ATCCGGCACCGCTGAAGGCTGCG | *mexC_1,2_* mRNA quantification |
| *mexC_1,2_*-R | CGGATCGAGCTGCTGGATGCGCG |  |
| *mexC_3,4_*-F | GTACCGGCGTCATGCAGGGTTC | *mexC_3,4_* mRNA quantification |
| *mexC_3,4_*-R | TTACTGTTGCGGCGCAGGTGACT |  |
| *16s rRNA*-F | CGGTGAATACGTTCYCGG | *16s rRNA* quantification |
| *16s rRNA*-R | GGWTACCTTGTTACGACTT |  |

F, forward primer; R, reverse primer

**Table S2. MICs of antibiotics against *P. aeruginosa* surviving UV irradiation**

| Antibitotics |  | MIC (mg/L) | | | |
| --- | --- | --- | --- | --- | --- |
|  | UV dose (mJ/cm^2^) | 0 | 5.50 | 8.25 | 11.00 |
| tetracycline |  | 8 | 8 | 8 | 16 |
|  |  | 8 | 8 | 16 | 16 |
|  |  | 8 | 8 | 16 | 16 |
|  |  | 8 | 8 | 16 | 32 |
| ciprofloxacin |  | 0.125 | 0.25 | 0.25 | 0.25 |
|  |  | 0.125 | 0.25 | 1 | 1 |
|  |  | 0.125 | 0.25 | 1 | 1 |
|  |  | 0.125 | 0.25 | 1 | 2 |
| polymyxin B |  | 8 | 8 | 8 | 16 |
|  |  | 8 | 8 | 8 | 16 |
|  |  | 8 | 8 | 8 | 16 |
|  |  | 8 | 8 | 8 | 16 |
| ceftazidime |  | 0.25 | 0.25 | 0.25 | 0.25 |
|  |  | 0.25 | 0.25 | 0.25 | 0.25 |
|  |  | 0.25 | 0.25 | 0.25 | 0.50 |
|  |  | 0.25 | 0.25 | 0.25 | 0.25 |
| chloramphenicol |  | 64 | 64 | 64 | 64 |
|  |  | 64 | 64 | 64 | 64 |
|  |  | 64 | 64 | 64 | 64 |
|  |  | 64 | 64 | 64 | 64 |
| gentamicin |  | 0.015 | 0.015 | 0.015 | 0.015 |
|  |  | 0.015 | 0.015 | 0.015 | 0.015 |
|  |  | 0.015 | 0.015 | 0.015 | 0.015 |
|  |  | 0.015 | 0.015 | 0.015 | 0.015 |

**Table S4. Summary of changes in gene expression probably related to antibiotic susceptibility in UV-exposed *P. aeruginosa***

| Gene name | Product | Fold change |
| --- | --- | --- |
| *mexC* | RND multidrug efflux membrane fusion protein MexC | 2.03 |
| *oprP* | Phosphate-specific outer membrane porin OprP | 2.21 |
| *PA4096* | major facilitator superfamily transporter | 3.97 |
| *PA3303* | major facilitator superfamily transporter | 3.63 |
| *PA1131* | major facilitator superfamily transporter | 2.91 |
| *PA2472* | major facilitator superfamily transporter | 2.47 |
| *PA3718* | major facilitator superfamily transporter | 2.32 |
| *PA1282* | major facilitator superfamily transporter | 2.28 |
| *PA3336* | major facilitator superfamily transporter | 2.17 |
| *PA1212* | major facilitator superfamily transporter | 2.02 |
| *PA2987* | lipoprotein-releasing system ABC transporter ATP-binding protein | 2.00 |

**Table S5. Changes of gene expression associated with oxidative stress in *P. aeruginosa* following UV exposure**

| Gene name | Product | Fold change |
| --- | --- | --- |
| *katB* | catalase | 3.13 |
| *katE* | catalase HPII | 2.97 |
| *sodM* | Superoxide dismutase | 3.29 |
